# Lavender- and lavandin-distilled straws: an untapped feedstock with great potential for the production of high-added value compounds and fungal enzymes

**DOI:** 10.1186/s13068-018-1218-5

**Published:** 2018-08-02

**Authors:** Laurence Lesage-Meessen, Marine Bou, Christian Ginies, Didier Chevret, David Navarro, Elodie Drula, Estelle Bonnin, José C. del Río, Elise Odinot, Alexandra Bisotto, Jean-Guy Berrin, Jean-Claude Sigoillot, Craig B. Faulds, Anne Lomascolo

**Affiliations:** 1 UMR1163 BBF Biodiversité et Biotechnologie Fongiques, INRA, Aix Marseille Univ, 13288 Marseille Cedex 09, France; 2 UMR408 SQPOV Sécurité et Qualité des Produits d’Origine Végétale, INRA, Université d’Avignon, 33 rue Louis Pasteur, 84029 Avignon, France; 3 UMR1319 MICALIS Microbiologie de l’Alimentation au Service de la Santé Humaine, PAPPSO, INRA, 78352 Jouy-en-Josas Cedex, France; 4 USC1408 AFMB Architecture et Fonction des Macromolécules Biologiques, INRA, 13288 Marseille, France; 5 UR 1268 BIA Biopolymères, Interactions, Assemblage, INRA, 44316 Nantes, France; 6 Department of Plant Biotechnology, IRNAS, CSIC, Avda. Reina Mercedes, 10, 41012 Seville, Spain

**Keywords:** Lavender and lavandin straws, Sugar and lignin, Terpenes and phenolics, Antioxidant, *Pycnoporus cinnabarinus*, Laccase, Lytic polysaccharide monooxygenase, Biorefinery

## Abstract

**Background:**

Lavender (*Lavandula angustifolia*) and lavandin (a sterile hybrid of *L. angustifolia* × *L. latifolia*) essential oils are among those most commonly used in the world for various industrial purposes, including perfumes, pharmaceuticals and cosmetics. The solid residues from aromatic plant distillation such as lavender- and lavandin-distilled straws are generally considered as wastes, and consequently either left in the fields or burnt. However, lavender- and lavandin-distilled straws are a potentially renewable plant biomass as they are cheap, non-food materials that can be used as raw feedstocks for green chemistry industry. The objective of this work was to assess different pathways of valorization of these straws as bio-based platform chemicals and fungal enzymes of interest in biorefinery.

**Results:**

Sugar and lignin composition analyses and saccharification potential of the straw fractions revealed that these industrial by-products could be suitable for second-generation bioethanol prospective. The solvent extraction processes, developed specifically for these straws, released terpene derivatives (e.g. τ-cadinol, β-caryophyllene), lactones (e.g. coumarin, herniarin) and phenolic compounds of industrial interest, including rosmarinic acid which contributed to the high antioxidant activity of the straw extracts. Lavender and lavandin straws were also suitable inducers for the secretion of a wide panel of lignocellulose-acting enzymes (cellulases, hemicellulases and oxido-reductases) from the white-rot model fungus *Pycnoporus cinnabarinus.* Interestingly, high amounts of laccase and several lytic polysaccharide monooxygenases were identified in the lavender and lavandin straw secretomes using proteomics.

**Conclusions:**

The present study demonstrated that the distilled straws of lavender and lavandin are lignocellulosic-rich materials that can be used as raw feedstocks for producing high-added value compounds (antioxidants, aroma) and fungal oxidative enzymes, which represent opportunities to improve the decomposition of recalcitrant lignocellulose into biofuel. Hence, the structure and the physico-chemical properties of these straws clearly open new perspectives for use in biotechnological processes involving especially filamentous fungi. These approaches represent sustainable strategies to foster the development of a local circular bioeconomy.

**Electronic supplementary material:**

The online version of this article (10.1186/s13068-018-1218-5) contains supplementary material, which is available to authorized users.

## Background

*Lavandula* species (*Lamiaceae* family) are mainly grown for their essential oils which are used in perfumes, cosmetics, food processing and aromatherapy products. Three species are cultivated: fine lavender (*Lavandula angustifolia*), the most common one, spike lavender (*Lavandula latifolia*) and lavandin (*Lavandula x intermedia*), a sterile hybrid of *L. angustifolia* × *L. latifolia*. The aromatic qualities of lavender and lavandin are mainly due to the volatile compounds found in their essential oil, identified as terpenes or terpenoids [1]. Global production of lavender and lavandin essential oils is estimated at 200 and 1000 tons per year, respectively. Production is located in Europe, Ukraine, Asia and Northern Africa, but dominated by the South of France, which holds a 90% share of the global market for lavandin essential oil [2].

The essential oil is usually obtained from raw plant materials (flowers, buds, stems and leaves) by steam distillation, but yields are low with only 2–10% dry matter [1–3]. Consequently, large amounts of solid residues, estimated at around 20,000 tons (dry matter) of lavandin- and lavender-distilled straws (LLDS), are generated every year in France [4]. These LLDS residues can be an environmental concern if they are not properly managed, but they are also a significant source of bioactive compounds that can increase the overall profitability of the aromatic plant.

Lavender- and lavandin-distilled straws are generally considered as waste materials and have traditionally been burnt to generate energy or used for composting. However, recycling LLDS by composting carries disadvantages due to the anti-germinative properties of certain components [5]. New alternatives to these traditional uses have emerged that make use of the physicochemical properties and composition of LLDS. Distilled lavender straw was successfully recycled as bio-based aggregates for building materials due to its hygrothermal properties [6]. In addition, many *Lamiaceae* plants are known to contain a wide range of phenolic compounds with antioxidant activity [7]. As phenolic compounds like hydroxycinnamic acids and flavonoids are not volatile and not degraded by thermal treatment [8], they remain in the waste material after distillation. For instance, antioxidant phenolic compounds, including rosmarinic acid, apigenin and luteolin, were found in spike lavender residues and lavandin wastes [5, 9].

A basic chemical composition analysis (nitrogen, carbon, hydrogen, oxygen and sulfur), by the French Inter-Regional Center for Experimentation in Medicinal and Aromatic Plants [4] revealed that the LLDS were carbon-rich residues (around 50% dry matter), originating especially from lignocellulose [2, 10], the most abundant biopolymer on Earth composed of carbohydrate polymers (cellulose, hemicellulose) and recalcitrant aromatic polymer (lignin) [11]. From a biotechnological point of view, LLDS are a largely untapped biomass resource for sustainable chemistry.

Filamentous fungi are microorganisms that can thrive on a wide variety of lignocellulosic by-products for biotransformation or delignification, including cereal brans, sugar beet pulp, rapeseed and sunflower meal, wheat straw [12–14]. This ability of fungi to degrade lignocellulosic materials is due to their highly efficient and extracellular enzymatic systems: (i) a huge hydrolytic arsenal, which is used, for instance, for the saccharification of lignocellulosic residues generating (after fermentation) second-generation bioethanol [15, 16], and (ii) a unique oxidative and lignolytic system, which degrades and modifies aromatic compounds including lignin [17]. In a green chemistry framework, the use of filamentous fungi may be attractive for developing new LLDS valorization routes, given their lignocellulose-degrading enzyme machinery.

The general objective of this work was to assess the potential of LLDS as a feedstock for high-added value bio-based compounds and fungal enzyme production biotechnological processes. LLDS, fractionated into stem straws and flower straws were analyzed in terms of composition and their saccharification potential and antioxidant activity were assessed. The utilization of each fraction of lavender and lavandin straws as substrate for enzyme production and growth of the model white-rot fungus *Pycnoporus cinnabarinus* was investigated using proteomics. This study opens new prospects for the use of lavender and lavandin by-products in white biotechnology.

## Results

### Lignocellulose composition of lavender and lavandin straws and saccharification potential

The distribution of lignin and polysaccharides in the LLDS fractions is shown in Table 1. The DM content was in the range 92–93%. Lignin and cellulose amounts were comparable between lavandin and lavender straws, while the composition of stem straws (DS and LS) and flowers straws (DF and LF) differed. The acid-insoluble lignin content was slightly lower in DS and LS (~ 25% DM) than in DF and LF (~ 29% DM). Cellulose content was two-fold higher in DS and LS (~ 16–17% DM) than in DF and LF (~ 8% DM), whereas hemicellulose and pectin contents were similar for stem and flower straws. Cell wall polysaccharide’s composition was broadly similar between lavandin and lavender straws. All the fractions yielded rhamnose, mannose, galactose, arabinose, glucose, xylose and galacturonic acids, although the relative amounts of each monomer varied from fraction to fraction. The rhamnose, arabinose, galactose and galacturonic acid contents, mainly pectin constituents, were higher in flower straws than in stem straws. Conversely, xylose content was higher in stem straws than in flower straws.

**Table 1:** Composition of lavandin- and lavender-distilled straws, expressed as percentage of dry matter

	Lavandin		Lavender	
	DS	DF	LS	LF
Moisture	6.85 ± 0.05	6.65 ± 0.28	7.15 ± 0.49	7.60 ± 0.49
Acid-insoluble lignin	25.64 ± 0.33	29.42 ± 0.57	24.99 ± 0.34	29.22 ± 0.48
Hemicelluloses and pectins	29.81 ± 0.59	30.44 ± 0.06	27.82 ± 0.19	26.84 ± 0.07
Rhamnose^a^	0.36 ± 0.01	0.47 ± 0.06	0.42 ± 0.01	0.52 ± 0.01
Fucose^a^	0	0	0	0
Arabinose^a^	1.8 ± 0.03	3.50 ± 0.03	1.56 ± 0.14	2.58 ± 0.01
Xylose^a^	14.20 ± 0.45	5.59 ± 0.22	13.14 ± 0.20	6.28 ± 0.13
Mannose^a^	1.06 ± 0.02	1.00 ± 0.01	1.15 ± 0.02	1.05 ± 0.11
Galactose^a^	1.32 ± 0.06	2.94 ± 0.06	1.35 ± 0.03	2.16 ± 0.22
Glucose^a^	4.51 ± 0.11	6.39 ± 0.02	3.16 ± 0.01	4.47 ± 0.12
Galacturonic acids^a^	6.58 ± 0.00	10.56 ± 0.00	7.05 ± 0.00	9.80 ± 0.00
Cellulose	16.06 ± 0.50	7.95 ± 0.03	17.49 ± 0.06	8.23 ± 0.46

The experiments were performed in duplicate (*n* = 2)

*DS* lavandin stem straw, *DF* lavandin flower straw, *LS* lavender stem straw, *LF* lavender flower straw

^a^Monomer analysis after hydrolysis (see “Methods”)

The lignin composition of the LLDS fractions (DS, DF, LS and LF) was characterized by Py–GC/MS. Pyrolysis produces a thermal breakdown of the lignin polymer into monomeric fragments that can be readily analyzed by GC–MS and is a useful tool to determine lignin composition in terms of the *p*-hydroxyphenyl (H), guaiacyl (G) and syringyl (S) lignin units and the S/G ratios of plant samples [18]. In all cases, the pyrograms of the LLDS fractions mostly showed compounds derived from carbohydrates, terpenoids and proteins, whereas the lignin-derived compounds were present in lower amounts. Among the lignin-derived compounds, the pyrograms showed compounds derived from G-lignin units such as guaiacol, 4-methylguaiacol, 4-ethylguaiacol, 4-vinylguaiacol and *trans*-4-propenylguaiacol, and compounds derived from S-lignin units such as syringol, 4-methylsyringol, 4-ethylsyringol, 4-vinylsyringol and *trans*-4-propenylsyringol (Table 2). The lignin composition of the LLDS fractions (in terms of their S/G ratios) was estimated from the peak areas of the respective compounds in the pyrograms and indicated that the stem straws (DS and LS) presented similar amounts of G- and S-lignin units (S/G ratios of 0.9), whereas the flower straws (DF and LF) were highly enriched in G-lignin units (S/G ratios of 0.2–0.3).

**Table 2:** Selected G- and S-lignin-derived compounds released upon Py–GC/MS of the different samples, and S/G ratios

	Lavandin		Lavender	
	DS	DF	LS	LF
Py–GC/MS analysis^a^				
Guaiacyl-derived compounds				
Guaiacol	31.4	28.0	39.3	13.1
4-Methylguaiacol	11.6	11.2	9.52	4.33
4-Ethylguaiacol	5.48	6.10	6.11	2.39
4-Vinylguaiacol	53.5	42.0	56.3	19.3
4-Propenylguaiacol	23.5	16.1	22.0	7.52
Syringyl-derived compounds				
Syringol	32.3	6.17	42.1	5.60
4-Methylsyringol	11.1	1.68	9.31	1.39
4-Ethylsyringol	6.23	1.17	7.59	0.88
4-Vinylsyringol	40.1	4.39	40.3	4.61
4-Propenylsyringol	20.7	2.15	20.6	2.50
S/G ratio	0.9	0.2	0.9	0.3

*DS* lavandin stem straw, *DF* lavandin flower straw, *LS* lavender stem straw, *LF* lavender flower straw

^a^Data are given as peak areas (× 10^−6^) and S/G ratios were calculated based on the peak areas

**Fig. 1:**
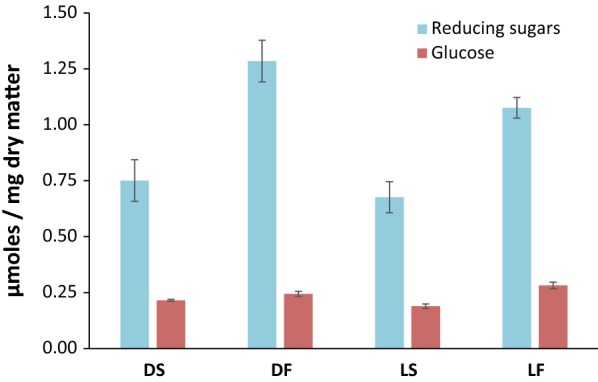
Saccharification assays of lavandin- and lavender-distilled straws using *T. reesei* E508 enzymatic cocktail. The reducing sugars and glucose released from the residues were expressed as glucose equivalent (GluE) in µmole per mg of dry matter. Values are given at 6 h and are means of triplicate. Standard errors of the mean were less than 5%

Saccharification of DS, DF, LS and LF fractions was performed using a *Trichoderma reesei* enzymatic cocktail, containing mainly cellulase activity [15, 19]. The results after 6 h of hydrolysis, expressed as glucose equivalents (GluE) in µmol per mg DM, are summarized on Fig. 1. The *T. reesei* cocktail enabled effective release of reducing sugars and glucose. It is worth noting that the release of reducing sugars was 1.7 times higher for the DF and LF fractions (1.2 µmol GluE/mg on average) than for DS and LS fractions (0.72 µmol GluE/mg on average). Similarly, glucose release was 1.3 times higher for DF and LF fractions (0.28 µmol/mg on average) than DS and LS fractions (0.21 µmol/mg on average). In addition, the release of reducing sugars and glucose was 1.2 times more effective for lavender than for lavandin-distilled straws. As a benchmark, the same *T. reesei* cocktail enabled the release of 0.98 µmol GluE from wheat straw and 0.26 µmol GluE from spruce, after 24 h of incubation [15]. The steam distillation of lavender and lavandin may have functioned as pretreatment, and this may partially explain why the saccharification was higher than that of wheat straw and spruce.

### Phenolic and terpene content of LLDS and antiradical activity

The total phenolic content (expressed as chlorogenic acid equivalents) of the ethanol and ethyl acetate extracts of LLDS was determined for the distilled straw fractions, DS, DF, LS and LF (Table 3). The yield of phenolic compound recovery by ethyl acetate was around 30–35%. In the ethyl acetate extracts, total phenolic content was higher for lavandin fractions, DS and DF (0.77 and 0.79 g ChAE/100 g DM, respectively) than for lavender fractions, LS and LF (0.60 and 0.62 g ChAE/100 g DM, respectively). These results were consistent with those of a recent study [9], resulting in a total phenolic content of around 0.8 g GAE (gallic acid equivalent) in the ethyl acetate fraction per 100 g of lavandin waste (DM). Stem and flower straws were not separated in that case.

**Table 3:** Total phenolic content of ethanolic and ethyl acetate extracts from distilled straws

	Lavandin		Lavender	
	DS	DF	LS	LF
Ethanolic extract				
^a^ChAE g/100 g dry mass	2.15 ± 0.07	2.25 ± 0.21	2.05 ± 0.21	2.30 ± 0.22
Ethyl acetate extract				
^a^ChAE g/100 g dry mass	0.77 ± 0.01	0.79 ± 0.02	0.60 ± 0.01	0.62 ± 0.06

The experiments were performed in duplicate (*n* = 2)

*DS* lavandin stem straw, *DF* lavandin flower straw, *LS* lavender stem straw, *LF* lavender flower straw

^a^ChAE: chlorogenic acid equivalents (g/100 g dry mass of straw)

The terpene-enriched cyclohexane fractions and the phenolics-enriched ethyl acetate fractions were analyzed by GC–MS to identify the molecules present in the stems and flowers of LLDS (Table 4). A total of 28 terpene derivatives, lactones and phenolic compounds were identified. Eighteen compounds were detected in the cyclohexane extracts. Coumarin, herniarin, τ-cadinol and β-caryophyllene were common to all the LLDS fractions. Among the terpenes and terpenoids detected, seven were specific for lavandin-straw stems and lavender-straw flowers: β-myrcene, borneol, cis-geraniol, geranyl propionate, germacrene d, α-bisabolol and camphor. In addition, in this study, seven compounds were found to be common to DS, DF and LS, two monoterpenols (linalool and α-terpineol), four terpenic esters (dihydrocarvyl acetate, geranyl acetate, linalyl acetate and neryl acetate) and one sesquiterpene, β-farnesene. Our results are useful complements to previous studies [20], which found coumarin, dihydrocoumarin, herniarin, α and τ-cadinol, α-bisabolol and camphor in the cyclohexanic extract of distilled lavandin (cultivar Grosso) waste. In addition, ten phenolic compounds (mainly derivatives of benzoic and cinnamic acids) were detected in the ethyl acetate extracts. The molecules were identified in all the DS, DF, LS and LF fractions.

**Table 4:** Compounds identified by GC–MS in the cyclohexane and ethyl acetate fractions of distilled straws

	Compound nature	Compounds	MS fragments^a^ *m*/*z* (relative intensity, %)	Substrates^b^
Cyclohexane fraction	Monoterpenes	β-Myrcene	M^+c^ *136* (2), 41 (100), 93 (74), 69 (68), 39 (28), 27 (19), 91 (17)	DS, DF
	Monoterpenols	Borneol	M^+^ *154* (tr), 95 (100), 41 (32), 27 (21), 39 (20), 110 (20), 43 (18)	DS, DF
		Linalool	M^+^ *154* (tr), 41 (100), 71 (64), 43 (60), 55 (45), 93 (39), 69 (36)	DS, DF, LF
		*cis*-Geraniol (nerol)	M^+^ *154* (2), 41 (100), 69 (66), 39 (30), 93 (26), 53 (12), 68 (12)	DS, DF
		α-Terpineol	M^+^ *154* (tr), 59 (100), 43 (74), 93 (72), 121 (52), 68 (44), 136 (42)	DS, DF, LF
	Terpenic esters	Dihydrocarvyl acetate	M^+^ *196* (tr), 43 (100), 41 (59), 39 (39), 93 (31), 67 (28), 53 (25)	DS, DF, LF
		Geranyl acetate	M^+^ *196* (tr), 69 (100), 43 (67), 41 (65), 68 (38), 93 (22), 136 (17)	DS, DT, LF
		Geranyl propionate	M^+^ *210* (tr), 41 (100), 69 (99), 57 (76), 29 (70), 68 (54), 93 (42)	DS, DF
		Linalyl acetate	M^+^ *196* (tr), 43 (100), 93 (82), 41 (58), 80 (33), 69 (24), 55 (23)	DS, DF, LF
		Neryl acetate	M^+^ *196* (tr), 69 (100), 41 (70), 43 (58), 68 (40), 93 (36), 80 (18)	DS, DF, LF
	Sesquiterpenes	β-Caryophyllene	M^+^ *204* (tr), 41 (100), 79 (41), 91 (40), 39 (34), 53 (30), 77 (28)	DS, DF, LS, LF
		β-Farnesene	M^+^ *204* (2), 41 (100), 69 (74), 93 (37), 39 (26), 67 (22), 79 (19)	DS, DF, LS
		Germacrene d	M^+^ *204* (14), 161 (100), 91 (91), 41 (86), 105 (80), 79 (52), 81 (52)	DS, DF
	Sesquiterpenoids	α-Bisabolol	M^+^ *222* (tr), 43 (100), 41 (70), 69 (50), 119 (50), 109 (42), 67 (28)	DS, DF
		τ-Cadinol	M^+^ *222* (tr), 161 (100), 43 (99), 119 (56), 41 (51), 105 (47), 204 (34)	DS, DF, LS, LF
	Ketones	Camphor	M^+^ *152* (19), 95 (100), 41 (89), 81 (70), 39 (50), 55 (50), 69 (40)	DS, DF
	Lactones	Coumarin	M^+^ *146* (91), 118 (100), 90 (47), 89 (39), 63 (38), 39 (18), 62 (16)	DS, DF, LS, LF
		Herniarin (7-methoxycoumarin)	M^+^ *176* (85), 133 (100), 148 (69), 77 (22), 51 (22), 63 (18), 105 (17)	DS, DF, LS, LF
Ethyl acetate fraction	Phenolic acids and derivatives	Caffeic acid isomers	M^+^ *398* (20), 219 (100), 73 (94), 396 (92), 397 (38), 191 (22), 381 (21)	DS, DF, LS, LF
		Catechol lactate	M^+^ *486* (5), 267 (100), 73 (97), 179 (28), 268 (27), 147 (24), 396 (17)	DS, DF, LS, LF
		*o/m*-Coumaric acid	M^+^ *308* (24), 73 (100), 147 (67), 293 (31), 131 (31), 161 (27), 75 (24)	DS, DF, LS, LF
		*p*-Coumaric acid	M^+^ *308* (22), 73 (100), 75 (39), 219 (32), 293 (30), 249 (17), 147 (10)	DS, DF, LS, LF
		2-Hydroxybenzoic acid	M^+^ *282* (tr), 73 (85), 135 (35), 268 (26), 91 (14), 269 (12), 179 (8)	DS, DF, LS, LF
		3-(2-Hydroxyphenyl)propionic acid	M^+^ *310* (45), 73 (100), 147 (83), 192 (73), 295 (62), 177 (47), 253 (39)	DS, DF, LS, LF
		3-(4-Hydroxyphenyl)lactic acid	M^+^ *398* (tr), 73 (100), 179 (95), 147 (31), 308 (29), 180 (16), 75 (13)	DS, DF, LS, LF
		*trans*/*cis* Ferulic acid	M^+^ *338* (67), 73 (100), 204 (43), 75 (42), 323 (33), 308 (30), 249 (27)	DS, DF, LS, LF
		Protocatechuic acid	M^+^ *370* (46), 73 (100), 193 (89), 355 (23), 223 (18), 311 (17), 371 (15)	DS, DF, LS, LF
		Vanillic acid	M^+^ *312* (61), 297 (100), 73 (81), 267 (73), 223 (59), 253 (42), 75 (29)	DS, DF, LS, LF

*tr* traces

^a^MS fragments of compounds present in the ethyl acetate fraction were given as trimethylsilylated derivatives

^b^DS, lavandin stem straw; DF, lavandin flower straw; LS, lavender stem straw; LF, lavender flower straw

M^+^: molecular ion (in italic)

Quantitative analysis of aromatics found in the ethyl acetate extracts of LLDS was performed by HPLC after calibration with available commercial standards (Table 5). Only five molecules could be identified as their aglycones, (i) one phenolic acid, rosmarinic acid, an ester of caffeic acid, (ii) two flavonoids, luteolin and apigenin, tri-and tetra-hydroxylated, respectively, and (iii) two lactones, coumarin and herniarin. DF and LF were high-producing fractions of rosmarinic acid (reaching 78 and 67 mg/100 g DM, respectively) and, to a lesser extent, of luteolin and apigenin. Coumarin and herniarin were present almost in all the LLDS fractions and were especially abundant in lavender straws, with values of 27 and 44, and 23 and 74 mg/100 g DM for LS and LF, respectively.

**Table 5:** Aromatic compounds quantified in the ethyl acetate extracts of distilled straws

Compound	Lavandin		Lavender	
	DS	DF	LS	LF
Phenolics				
Rosmarinic acid	4 ± 0.2	78 ± 4	nd	67 ± 3
Luteolin	3 ± 0.1	11 ± 0.6	3 ± 0.2	6 ± 0.3
Apigenin	nd	17 ± 0.9	1 ± 0.1	9 ± 0.5
Lactones				
Coumarin	nd	25 ± 1.3	27 ± 1.4	44 ± 2.2
Herniarin	8 ± 0.4	23 ± 1.2	23 ± 1.3	74 ± 3.7

The amounts are expressed in mg per 100 g of dry matter

The experiments were performed in duplicate (*n* = 2)

*DS* lavandin stem straw, *DF* lavandin flower straw, *LS* lavender stem straw, *LF* lavender flower straw, *nd* not detected

Antiradical scavenging activity, estimated as the inhibitory concentration IC_50_ and expressed as µg ChAE/mL of reaction mixture, was evaluated on the ethyl acetate extracts from the different fractions of LLDS. It was compared to the antiradical scavenging activity of the commercial synthetic antioxidant butylhydroxytoluene (BHT) and the two natural compounds (chlorogenic acid and rosmarinic acid) typically found in lavandin straws and known for their antioxidant activity [9], which were used as references (Table 6). For these reference molecules, the rank of antiradical activity, in decreasing order, was rosmarinic acid followed by chlorogenic acid and BHT. All the LLDS samples tested displayed a lower IC_50_ (i.e. higher antioxidant activity) than BHT. The ethyl acetate extracts, from both lavandin and lavender straws, showed a greater antiradical activity for flowers straws (LF and DF) than for stems straws (LS and DS) with values of 2.44–4.28 and 8.05–8.45 µg ChAE/mL, respectively.

**Table 6:** Antiradical activity of ethyl acetate extracts from distilled straws

Type of compounds or LLDS fraction	CI_50_ (µg ChAE/mL reaction mixture)
Rosmarinic acid	0.70 ± 0.05
Chlorogenic acid	3.19 ± 0.17
Butylhydroxytoluene (BHT)	15.17 ± 4.57
DS	8.05 ± 0.80
DF	2.44 ± 0.01
LS	8.45 ± 0.37
LF	4.28 ± 0.01

The experiments were performed in duplicate (*n* = 2)

*DS* lavandin stem straw, *DF* lavandin flower straw, *LS* lavender stem straw, *LF* lavender flower straw

### Secretomics of the fungus *P. cinnabarinus* grown on lavandin and lavender straws

Lavender- and lavandin-distilled straws fractions were compatible with the growth of *P. cinnabarinus* BRFM 137. Indeed, growth tests first performed on agar plates containing only lavandin and lavender straw as a carbon source did not show any toxicity of the different fractions for the fungus. Radial growth was not inhibited, being similar or better than on the reference medium containing only maltose (Additional file 1: Figure S1).

To determine the composition of the secretomes (secreted proteins) produced by *P. cinnabarinus* BRFM 137 during its growth on the LLDS fractions, we performed, by LC–MS/MS, proteomic analysis on the liquid culture supernatants after 3, 7 and 10 days of growth on each fraction and reference medium. Overall, up to 189 proteins were identified by mass-matching against a database derived from the genome annotation of the strain *P. cinnabarinus* BRFM 137 [21] (Table 7; Additional file 2: Data S1).

**Table 7:** Proteins (CAZymes) identified in the *P. cinnabarinus* secretomes

	Lavandin		Lavender		Reference
	DS	DF	LS	LF	Maltose
Number of total proteins detected	153	189	147	176	164
Number of predicted CAZymes	85	91	81	90	81
GH	56	58	56	61	56
PL	3	5	2	4	4
CE	7	8	6	7	6
AA	17	19	15	17	14
AA1_1 (laccases)	4	4	4	4	4
AA1_2 (ferroxidases)	1	1	1	1	0
AA2 (class II peroxidases, manganese peroxidase)	0	0	0	0	1
AA3_1 (cellobiose dehydrogenases)	1	1	1	1	1
AA3_2 (aryl alcohol oxidases/glucose oxidases)	2	3	2	4	4
AA3_4 (pyranose oxidases)	0	1	0	1	1
AA5_1 (copper radical oxidases/glyoxal oxidases)	2	2	2	2	4
AA6 (benzoquinone reductases)	0	1	0	1	0
AA9 (lytic polysaccharide monooxygenases)	7	6	5	4	0
Number of non-CAZy proteins	70	99	68	87	84

*DS* lavandin stem straw, *DF* lavandin flower straw, *LS* lavender stem straw, *LF* lavender flower straw, *GH* glycosyl hydrolases, *AA* auxiliary activities, *PL* polysaccharide lyases, *CE* carbohydrate esterases

Due to the high lignocellulose content of LLDS, a special focus was given to the carbohydrate-active enzymes (CAZymes), that encompass enzymes dedicated to the modification and breakdown of plant cell wall polysaccharides [22, 23], and on “auxiliary activities” enzymes (AA, redox enzymes). A large diversity of CAZymes was identified in fungal secretomes with about 50–55% of the total proteins (Table 7; Additional file 1: Figure S2). To a lesser extent, proteins with predicted proteolytic activity, related to amino acid and lipid metabolism were also identified (Additional file 1: Table S1, Additional file 2: Data S1). The secretome composition slightly differed according to the nature of the LLDS fraction. The DS and LS secretomes have slightly higher proportions of glycoside hydrolases (GHs), carbohydrate esterases (CEs) and AAs than the other secretomes, (Table 7; Additional file 3: Data S2). The AA class encompasses a large group of lignolytic and polysaccharide oxidases, including various enzymes of industrial interest [22]. Interestingly, the secretomes of *P. cinnabarinus* BRFM 137 contained between 14 and 19 different AAs (Table 7), distributed across the (sub)families AA1_1, AA1_2, AA2, AA3_1, AA3_2, AA3_4, AA5_1, AA6 and AA9. Interestingly, AA1_2, AA6, and AA9, were only detected in the LLDS secretomes (Table 7; Additional file 3: Data S2), while AA3_4 (a predicted function of pyranose oxidase) and AA6 (a predicted function of benzoquinone reductase) were only found in the DF-and LF-secretomes. Enzymes from the AA9 family correspond to lytic polysaccharide monooxygenases (LPMOs), which boost the activity of GHs through the oxidative cleavage of recalcitrant polysaccharides. Out of the 16 AA9 LPMOs encoded by the *P. cinnabarinus* BRFM137 genome, seven were identified in LLDS secretomes. Two of them harbour a CBM1 module that might potentiate their activity on cellulose. Interestingly, the *P. cinnabarinus* cellobiose dehydrogenase (CDH, AA3_1-AA8) providing electron to the LPMOs is also present in LLDS secretomes.

### Production of laccase by *P. cinnabarinus* in the presence of LLDS

**Fig. 2:**
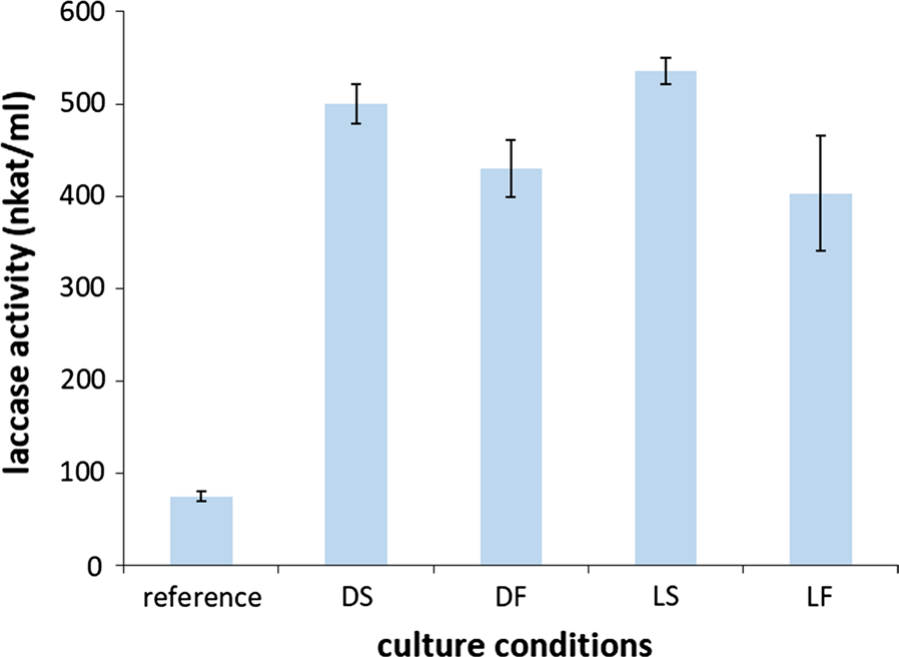
Laccase activity in the culture broths of *P. cinnabarinus.* The fungus was grown on natural substrates (DS, LS, DF, LF), or maltose (reference)

Among the extracellular proteins identified in the secretomes of *P. cinnabarinus* grown in the presence of LLDS, AA1_1 laccases (*p*-diphenol:oxygen oxidoreductases) were amongst the most abundant proteins secreted. Laccases are multicopper enzymes of industrial interest for white biotechnology [24, 25]. To further investigate the production of laccase, we monitored the laccase activity for 14 days in cultures induced by DS, DF, LS and LF (Fig. 2). Maximum laccase activity (400–540 nkat/mL) was obtained on day 14 of culture, corresponding to 85–125 mg/mL of enzyme (specific activity of 4330 nkat/mg [26]), which was 5–7 times higher than the activity obtained in the control medium (75 nkat/mL in the absence of any inducer) and 1.5–2 times higher than that obtained in the presence of ferulic acid (250 nkat/mL on average), a phenolic acid conventionally used as laccase inducer [27]. Among the conditions tested, the LS fraction was the most suitable inducer for high laccase production.

## Discussion

The solid residues obtained after essential oil distillation from lavender and lavandin are generally considered as wastes. This work allowed characterizing lavender- and lavandin-distilled straws as a potentially renewable plant biomass and a source of biomolecules. Diverse ways of valorization were assessed for value recovery.

The cell wall composition of LLDS was determined after fractionation into residual stems, described as a straw-like structure, and residual flowers, the surface of which harbors glandular structures storing essential oil in *Lamiaceae* [3]. While the straws of lavandin and lavender displayed similar amounts of lignin and cellulose, hemicellulose and pectin contents were higher in lavandin than in lavender straws. In agreement with the literature [10], we found two-fold lower cellulose content in flower straws than in stem straws. Lignin composition of each fraction of lavandin and lavender straws indicated that the flowers, whatever the species (lavandin or lavender) were highly enriched in G-lignin units (S/G ratios of 0.2–0.3), while the stem straws had a similar content of both G- and S-lignin units (S/G ratios of 0.9). G-rich lignin produces more condensed linkages because the C-5 position is free to have additional carbon–carbon linkages, thus increasing the recalcitrance of the lignin polymer. More generally, high S/G ratios (at least > 2) can facilitate the enzymatic degradation of the biomass and reduce recalcitrance [28]. Compared to wheat straw and spruce, the saccharification potential of lavender and lavandin straws is promising, considering that the hydrolysis stage can be improved by supplementing the *T. reesei* cocktail with additional fungal enzymes [15, 29].

The LLDS fractions contained interesting volatile molecules including terpenes and terpenoids, basically found in the essential oils, as well as non-volatile phenolic compounds and flavonoids which remained in the straws after distillation. Specific solvent extractions coupled with GC–MS and LC analyses enabled us to detect terpene derivatives, lactones and phenolics. Four molecules with potential industrial interest were identified in each fraction of distilled straws: (i) the sesquiterpene, τ-cadinol, known to have smooth muscle-relaxing properties [30], (ii) the sesquiterpene β-caryophyllene, which has significant anticancer and analgesic activities [31], and (iii) coumarin and herniarin, which are lactones possessing anti-inflammatory, antioxidant, antiviral, antimicrobial activities, and anti-spasmodic properties [20, 32, 33]. A wide variety of terpenes and terpenoids, such as linalool, linalyl acetate, borneol, α-terpineol and geraniol, all characteristic constituents of lavender and lavandin essential oils, was also recovered from the distilled straws and is known to have sedative, anesthetic, antispasmodic or antimicrobial actions [2]. Vanillic, coumaric and ferulic acids, known as precursors of bioconversion pathways for high-value molecules by filamentous fungi [25], were identified in each LLDS fraction. Chlorogenic acid, glycosylated conjugates of caffeic, ferulic and coumaric acids, and flavonoids (isoquercitrin and luteolin) with strong antioxidant properties have been already identified in lavandin waste [9]. Here, our LC protocol for phenolic compound analysis allowed the identification of rosmarinic acid as the major phenolic compound in the straws, contributing actively to the high radical scavenging capacity of LLDS, as previously reported in the spike lavender by-product obtained after essential oil distillation [5]. Phenolic compounds sharing the *ortho*-diphenol structure (including caffeic acid or derivatives) are known to be strong antioxidants [34]. Such natural molecules with strong antioxidant activity are progressively replacing synthetic antioxidants such as BHT, whose use is more limited due to possible adverse effects on human health [35]. The extraction of natural antioxidants from agricultural by-products is thus an opportunity to obtain profit from low-cost raw material, such as LLDS, in a sustainable way.

Today, we know that most agricultural by-products (e.g. sugar beet pulp, sugarcane bagasse, oilseed meals) are reliable sources of sugars and/or proteinaceous nutrients and can serve as a support matrix for various biotechnological processes including the fermentative production of enzymes [13]). In microbial, and especially filamentous fungal utilization, agricultural by-products can be used either as a growth carbon substrate and support in solid-state fermentation or as supplement to submerged production media. Here, we showed that LLDS were very good inducers for the production of a large array of lignocellulose-acting enzymes by the model fungus *P. cinnabarinus*, known for its ability to secrete a large panel of hydrolases and oxidoreductases [25]. In particular, LLDS were the best natural laccase inducers known to date for *P. cinnabarinus*; this laccase is a high-redox-potential enzyme already known for its versatility and efficiency in various biotechnological applications [25]. The concomitant induction of a CDH and several AA9 LPMOs, potentially targeting cellulose, supports a scenario where multiple AA9 LPMOs are required to mediate the decomposition of recalcitrant lignocellulose [36]. Some of these AA9 LPMOs were already detected in the secretomes of *P. cinnabarinus* cultivated on wheat straw [37] or on a mixture of birchwood, cellulose and autoclaved maize bran [21].

## Conclusions

The present study demonstrated that the distilled straws of lavender and lavandin are cheap and readily available industrial by-products of interest for producing high-added value compounds such as platform molecules (e.g. antioxidants) and fungal enzymes involved in the degradation of lignocellulosic biomass. These approaches could represent sustainable strategies to foster the development of a local circular bioeconomy.

## Methods

### Chemicals

All the chemicals used for the antioxidant measurements (DPPH, 1,1-diphenyl-2-picrylhydrazyl radical) and the total phenol assay (Folin–Ciocalteu reagent), and the phenolic standards (chlorogenic, rosmarinic, caffeic, *p*-coumaric, ferulic, vanillic and protocatechuic acids, and vanillin, luteolin, apigenin, coumarin, herniarin) were purchased from Sigma-Aldrich (Saint-Quentin Fallavier, France). Ethanol, ethyl acetate, cyclohexane, and acetonitrile were of HPLC grade and provided by Fisher Scientific (Illkirch-Graffenstaden, France).

### Raw materials

Distilled straws from fine lavender and the Super cultivar of lavandin were provided by the company Bontoux SA (Saint Auban sur l’Ouvèze, France). They were collected at the end of the harvest season (September 2013) just after the steam distillation process and then air-dried at room temperature in the laboratory for 10 days. Dried straws were separated into four fractions: lavandin stems (DS), lavandin flowers (DF), lavender stems (LS) and lavender flowers (LF). Before use, these fractions were ground to obtain particles sized 0.18–0.8 mm (IKA^®^ A11 knife mill, Werke GmbH & Co, Staufen, Germany). The dry matter content of each fraction DS, DF, LS and LF was measured by drying until constant mass at 105 °C.

### Fungal strain

The *P. cinnabarinus* strain used in this study was obtained from the fungal culture collection of the International Centre of Microbial Resources (CIRM-CF) at the French National Institute for Agricultural Research (INRA, Marseille, France). The strain was maintained on malt agar slants at 4 °C, using MA2 (malt extract broth Difco^®^ at 2% w/v) as medium, under the accession number BRFM 137.

### Culture conditions and secretome preparation

*Pycnoporus cinnabarinus* strain BRFM 137 was grown on a basal maltose medium [27]. Inoculum was obtained from precultures grown for 10 days at 30 °C in Roux flasks containing 180 mL of the following basal medium: maltose (20 g/L), diammonium tartrate (1.84 g/L), disodium tartrate (2.3 g/L), KH_2_PO_4_ (1.33 g/L), CaCl_2_·2H_2_O (0.1 g/L), MgSO_4_·7H_2_O (0.5 g/L), ZnSO_4_·7H_2_O (0.046 g/L), MnSO_4_·H_2_O (0.035 g/L), CuSO_4_·5H_2_O (0.007 g/L), yeast extract (1 g/L), and vitamin solution (1 mL/L) [38]. Mycelium from two flasks was collected, mixed with 50 mL sterile water, and blended for 1 min at 9000 rpm with an Ultra-Turrax T-25 system (Janke & Kunkel, GMBM & Co., KG, Staufen, Germany). A 5-mL sample of this suspension was inoculated into basal medium supplemented with 15 g/L of autoclaved straws (DS, DF, LS or LF) and 0.05 g/L of CuSO_4_·5H_2_O. Mycelial inoculum corresponded to 0.6–0.7 g mycelium dry mass (DM) per L of culture medium. Incubation was carried out in the dark at 30 °C and 120 rpm (Infors HT, Switzerland) in 250-mL baffled Erlenmeyer flasks containing 100 mL medium, for 10–14 days. Assays were carried out in triplicate.

Cultures were stopped after 3, 7 and 10 days of incubation. Based on previous work [39], the culture broths (secretomes) were separated from the mycelium by filtration through Miracloth™ paper, centrifuged at 3000 rpm for 15 min at 4 °C (Sorvall ST40R, Thermoscientific) and frozen at − 20 °C until use. The culture supernatants were pooled, filtered (using 0.22 μm polyethersulfone membranes, Vivaspin, Sartorius), concentrated (Vivaspin with a 5 kDa cut-off polyethersulfone membrane, Sartorius) to a final concentration of 1 mg/mL, and diafiltered with a 25 mM acetate solution buffer, pH 5. Total amount of protein was assessed using the Bradford assay (Bio-Rad Protein Assay Dye Reagent Concentrate, Ivry, France) with a BSA standard that ranged from 0.2 to 1 mg/mL.

### Identification of proteins by LC–MS/MS analysis

Proteins from the diafiltered supernatants of *P. cinnabarinus* BRFM 137 cultures, grown on DS, DF, LS, LF or maltose (reference) were separated by one-dimensional (1D) SDS-PAGE electrophoresis [39, 40]. After protein trypsinolysis, peptide analysis was performed by LC–MS/MS [40], at the PAPPSO facility platform (Plateforme d’Analyse Protéomique de Paris Sud-Ouest, Jouy-en-Josas, France). Based on the list of peptides, protein identification was performed by querying the MS/MS data against the predicted proteins obtained from the *P. cinnabarinus* BRFM 137 genome sequencing data [21], available via the Mycocosm-Joint Genome Institute Portal [41]. Functional annotations with GO, KEGG, KOG and SignalP were obtained from this portal. Proteins identified with at least two unique peptides and a log(*E*-value) lower than − 2.6 were validated. Expert CAZy annotations were performed according to the CAZy database [22, 23, 42], at the UMR7257 (Architecture et Fonction des Macromolecules Biologiques, Centre National de la Recherche Scientifique, Aix-Marseille Université, Marseille, France).

### Laccase assay

Laccase activity was determined daily in culture supernatants of *P. cinnabarinus* BRFM 137 grown on LLDS throughout the incubation period (up to 14 days) by monitoring A_420_ (extinction coefficient: 36,000 L/mol cm) with respect to the rate of oxidation of 500 µmol/L 2,2′-azino-bis-[3-ethylthiazoline-6-sulfonate] (ABTS) in a 50 mmol/L sodium tartrate buffer pH 4 at 30 °C [24]. Enzyme activity was expressed in nanokatals (nkat). One nanokatal of activity is defined as the quantity of enzyme causing the conversion of 1 nmol of substrate per second. The experiments were performed in triplicate, and the standard deviation was lower than 5% of the mean.

### Saccharification assays

The saccharification assays were performed via a previously described high-throughput automated method [43] using a Tecan Genesis Evo 200 robot (Tecan, Lyon, France). A 1% (w/v) suspension of each straw fraction (DS, DF, LS and LF) was prepared in 50 mM acetate buffer, pH 5, supplemented with 40 µg/mL of tetracycline as antibiotic and 30 µg/mL of cycloheximide as antifungal agent. After rehydration overnight at 4 °C, the resulting suspensions were robot-dispensed into 96-well plates and the plates were frozen at − 20 °C until needed. Saccharification was performed after addition of 30 µg of *Trichoderma reesei* E508 enzymatic cocktail produced from *T. reesei* CL847 strain (obtained from IFPEN, Rueil-Malmaison, France) at 37 °C with 8 Hz shaking. *T. reesei* enzyme cocktail contained 0.12 U of filter paper activity, 0.33 U of CMCase, 0.2 U of β-glucosidase, 1.6 U of xylanase, 0.02 U of mannanase and 0.02 U of arabinofuranosidase per mg of total protein [15]. A substrate-free negative control was set up by filling wells with 50 mM sodium acetate buffer, pH 5, and the background of soluble sugars present in the samples was determined by incubating them in the absence of E508 enzymatic cocktail. After 3 and 6 h of incubation, the saccharification reaction mixtures were filtered and recovered, the reducing sugars were quantified using the 3,5-dinitrosalicylic acid (DNS) method, and glucose content was measured using a Glucose RTU kit (Biomérieux, Marcy l’Etoile, France) following the manufacturer’s instructions. The results were expressed as µmol glucose equivalent (GluE)/mg DM. All the reactions were performed independently at least in triplicate.

### Analytical methods

Acid-insoluble lignin content of the straw fractions was determined gravimetrically according to the NREL procedures [44]. After pre-hydrolysis with 72% (w/w) H_2_SO_4_ for 1 h at 30 °C, samples were hydrolyzed with 4% (w/w) H_2_SO_4_ for 1 h at 120 °C and filtered over AP40 Millipore glass-fiber filters. The washed residue was dried (105 °C, overnight), and weighed to give acid-insoluble lignin.

Neutral sugar composition determination was performed as previously described [45]. The samples were freeze-dried prior to analysis. The monosaccharides were quantified after pre-hydrolysis with 72% (w/w) H_2_SO_4_ at 25 °C for 30 min followed by hydrolysis in 2 N H_2_SO_4_ at 100 °C for 2 h and derivatization into their alditol acetates which were then analyzed by gas chromatography (Auto–System, Perkin Elmer, Courtaboeuf, France). Chromatography conditions were as follows: silica capillary column BP-225 (25 m × 0.32 mm id, Chromoptic, Courtaboeuf, France), hydrogen as carrier gas at a constant temperature of 220 °C and a flow rate of 2.2 mL/min. Myo-inositol was used as internal standard.

Uronic acid content was determined according to the automated *m*-hydroxybiphenyl method using anhydrogalacturonic acid for calibration [46].

The recovery of phenolics present in LLDS was conducted with a two-step extraction procedure. DS, DF, LS and LF fractions (20 g wet mass) were first extracted by maceration with 500 mL of 80% (v/v) ethanol in water for 24 h at room temperature under gentle stirring. The ethanolic extract was subsequently clarified by filtration through Miracloth™ paper and Whatman^®^ GF/D glass-fiber filter (Merck Millipore, Fontenay-sous-Bois, France). An aliquot of the clarified ethanolic extract (50 mL) was adjusted to pH 3 with HCl and then extracted three times successively with the solvent system ethyl acetate/water/ethanol (2:3:1 v/v/v). The enriched phenolic ethyl acetate phases were pooled and dried with anhydrous sodium sulfate. The solvent was further evaporated to dryness and the extract was solubilized in either 2 mL of ethyl acetate for GC–MS analysis or 2 mL ethanol for the other subsequent analyses.

For terpene extraction, each distilled straw fraction (5 g wet mass) was extracted two-fold with 120 mL cyclohexane under reflux for 30 min. After evaporation of the solvent, the terpene extract was totally dried under nitrogen, weighed and dissolved in a minimum volume of cyclohexane for GC–MS analysis.

Total phenolic content was determined colorimetrically at 750 nm by the Folin–Ciocalteu reagent, using chlorogenic acid as calibration standard. Results of analyses were expressed as g chlorogenic acid equivalents (ChAE) per 100 g DM. The calibration curve was established from 0 to 100 mg/L chlorogenic acid.

The antiradical properties of DS, DF, LS and LF extracts were assessed as previously described [47]. Two milliliter of the sample to be tested was added to an ethanolic solution containing 60 µM DPPH. After 30 min in the dark, the absorbance was recorded at 517 nm and compared against the control (butylhydroxytoluene as synthetic antioxidant). Antiradical activity is defined as the sample concentration (IC_50_) needed to decrease the initial DPPH concentration by 50%. IC_50_ was expressed as µg ChAE per mL of reaction medium. The lower the IC_50_ value, the more potent the extract scavenges DPPH.

High-performance liquid-chromatography (HPLC) analysis of monophenolics was performed at 220 nm and 30 °C on a model Agilent1100 (Agilent Technologies, Massy, France) equipped with a variable UV/Vis detector and a 100-position autosampler/autoinjector. Separations were achieved on a C30 reversed-phase column (YMC™ Carotenoid 3 µm, 4.6 × 150 mm, Waters, Guyancourt, France). Flow rate was 0.8 mL/min. The mobile phases used were 0.05% phosphoric acid in water (solvent A) and acetonitrile 100% (solvent B). The gradient program was as follows: solvent B started at 15% for 5 min, increased to 40% in 15 min, then to 100% in 5 min until the end of the run (28 min). Quantification was performed by external standard calibration at concentrations ranging from 0 to 500 mg/L.

The qualitative analyses of samples were achieved using the gas chromatography–mass spectrometry (GC–MS) technique [48]. For phenolic analyses, the samples were submitted to the following procedure for derivatization prior to GC–MS analysis: 297 µL of *N*-methyl-*N*-(trimethylsilyl)fluoroacetamide (MSTFA) and 3 µL of 2% (*w/v*) methoxyamine hydrochloride in pyridine was incubated with the sample for 15 min at 60 °C. Trimethylsilyl derivatives of monomeric phenolic extracts were then analyzed by GC–MS using an Agilent 6890N GC-5973N mass detector (Agilent Technologies, Massy, France). GC–MS analyses of the terpene-rich cyclohexane extracts were performed without prior derivatization. The GC column was an Agilent DB5-MS (30 m × 0.25 mm i.d., 0.25 µm film thickness) with an inlet system using the 1:20 split injection technique. Injector temperature was 250 °C. Carrier gas was helium at a constant flow rate of 1 mL/min. For phenolic analyses, the oven temperature was held at 70 °C for 2 min, then raised to 280 °C at a rate of 10 °C/min and held at 280 °C for 5 min, then raised up to 300 °C at a rate of 10 °C/min, held at 300 °C for 5 min. Electron impact energy was set at 70 eV, ion source temperature was 230 °C and quadrupole temperature 150 °C. EI mass spectra ranged from 40 to 650 amu. For terpene analyses, the oven temperature was held at 50 °C for 1 min, then raised to 250 °C at a rate of 4 °C/min and held at 250 °C for 5 min. EI mass spectra ranged from 33 to 300 amu. Compounds were identified by comparing the mass spectra against those of the NIST library.

Samples (around 1 mg) were analyzed by pyrolysis–gas chromatography/mass spectrometry (Py–GC/MS) on a 3030 micro-furnace pyrolyzer (Frontier Laboratories Ltd., Fukushima, Japan) connected to an Agilent 7820A GC system using a DB-1701 fused-silica capillary column (60 m × 0.25 mm, 0.25 μm film thickness) and an Agilent 5975 mass selective detector (EI at 70 eV) (Agilent Technologies, Santa Clara, CA). Pyrolysis was performed at 500 °C. Temperature program of the GC oven was raised from 100 °C (4 min) to 280 °C (8 min) at 3 °C/min. The carrier gas was helium (1 mL/min). The compounds were identified by comparing their mass spectra against those of the Wiley and NIST libraries and those reported in the literature [49, 50], and when possible, by comparison with the retention times and mass spectra of authentic standards. Peak areas were calculated for the lignin-degradation products, and the ratio of the syringyl—to guaiacyl-derived compounds (S/G ratio) was determined.

## Additional files

**Additional file 1.** Additional Figures S1 and S2, and Table S1.

**Additional file 2: Data S1.** Excel table containing the list of proteins detected and identified in the secretomes of *P. cinnabarinus* BRFM137 grown on DS, DF, LS, LF or maltose (reference) as substrates.

**Additional file 3: Data S2.** Excel table containing the list of the Auxiliary Activities (AAs) detected and identified in the secretomes of *P. cinnabarinus* BRFM137 grown on DF, DS, LF, LS or maltose (reference) as substrates.

## Abbreviations

AA: auxiliary activity enzyme
ABTS: 2,2′-azino-bis-[3-ethylthiazoline-6-sulfonate]
BHT: butylhydroxytoluene
BRFM: Banque de Ressources Fongiques de Marseille, France
BSA: bovine serum albumin
CAZy: carbohydrate-active enzymes
CBM: carbohydrate-binding domain
CDH: cellobiose dehydrogenase
CE: carbohydrate esterases
EG: endoglucanase
GH: glycoside hydrolase
CIRM: International Centre of Microbial Resources
DM: dry matter
DF: the lavandin flower fraction obtained after fractionation of lavandin-distilled straws
DPPH^·^: 1,1-diphenyl-2-picrylhydrazyl radical
DS: the lavandin stem fraction obtained after fractionation of lavandin-distilled straws
G: guaiacyl unit
GC–MS: gas chromatography–mass spectrometry
H: hydroxyl unit
HPLC: high-performance liquid-chromatography
JGI: Joint Genome Institute (USA)
IC_50_: half-maximal inhibitory concentration
LC–MS/MS: liquid chromatography coupled with tandem mass spectrometry
LF: the lavender flower fraction obtained after fractionation of lavender-distilled straws
LLDS: lavender- and lavandin-distilled straws
LS: the lavender stem fraction obtained after fractionation of lavender-distilled straws
NIST: National Institute of Standards and Technology
NREL: National Renewable Energy Laboratory’s
PL: polysaccharide lyase
S: syringyl unit
SDS-PAGE: sodium dodecyl sulfate-polyacrylamide gel electrophoresis
