# Supplementary material for: Lavender- and lavandin-distilled straws: an untapped feedstock with great potential for the production of high-added value compounds and fungal enzymes
Source: Biotechnol Biofuels. 2018 Aug 2;11:217. doi: 10.1186/s13068-018-1218-5 (PMC6071384; doi:10.1186/s13068-018-1218-5)
Supplement: Supplementary file 1 — Additional file 1. Additional Figures S1 and S2, and Table S1. [file 13068_2018_1218_MOESM1_ESM.docx]

**Additional Figures:**


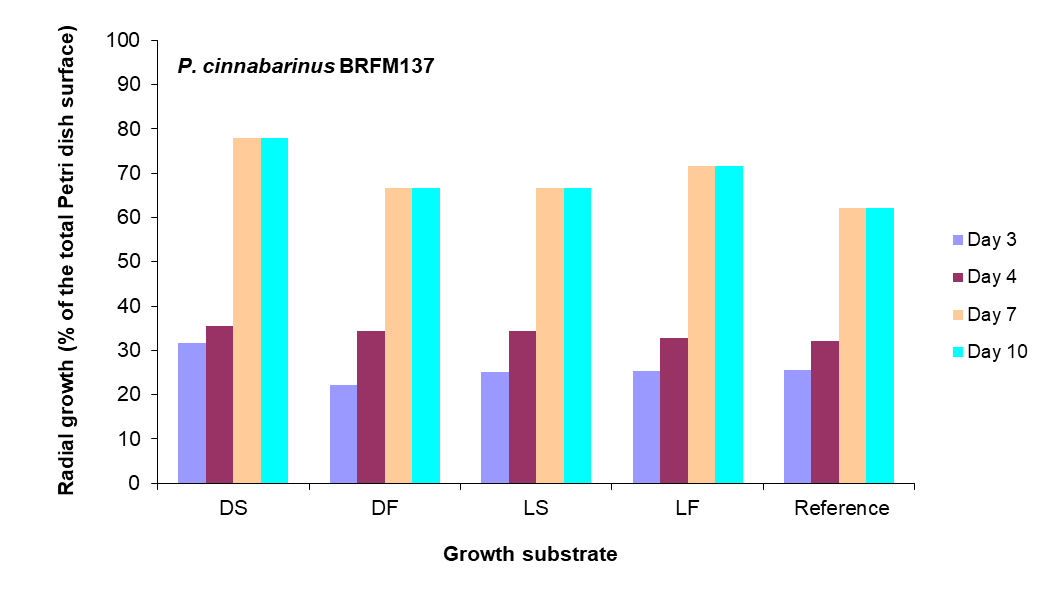


**Additional Figure S1 Evaluation of the toxicity of distilled straws on the mycelial radial growth of *P. cinnabarinus*.** Duplicate Petri dishes of each distilled straw fraction (DS, DF, LS and LF) used as carbon source were centrally inoculated by a 5-mm plug from an agar slant of the strain *P. cinnabarinus* BRFM137 and incubated at 30°C. Every day, the colony radial diameter was measured and compared between each culture condition. The increase in radial diameter was plotted as a function of the incubation time. The composition of the medium is the following: agar (15 g/L), straw substrate (DF, DS, LF or LS fraction) 20 g/L, Yeast Nitrogen Base w/o amino acids (Difco®) 1.7 g/L, (NH_4_)_2_C_4_H_4_O_6_ 1.84 g/L. The reference contained the same medium but depleted in straw substrates. DS: lavandin stem straw; DF: lavandin flower straw; LS: lavender stem straw; LF: lavender flower straw

**
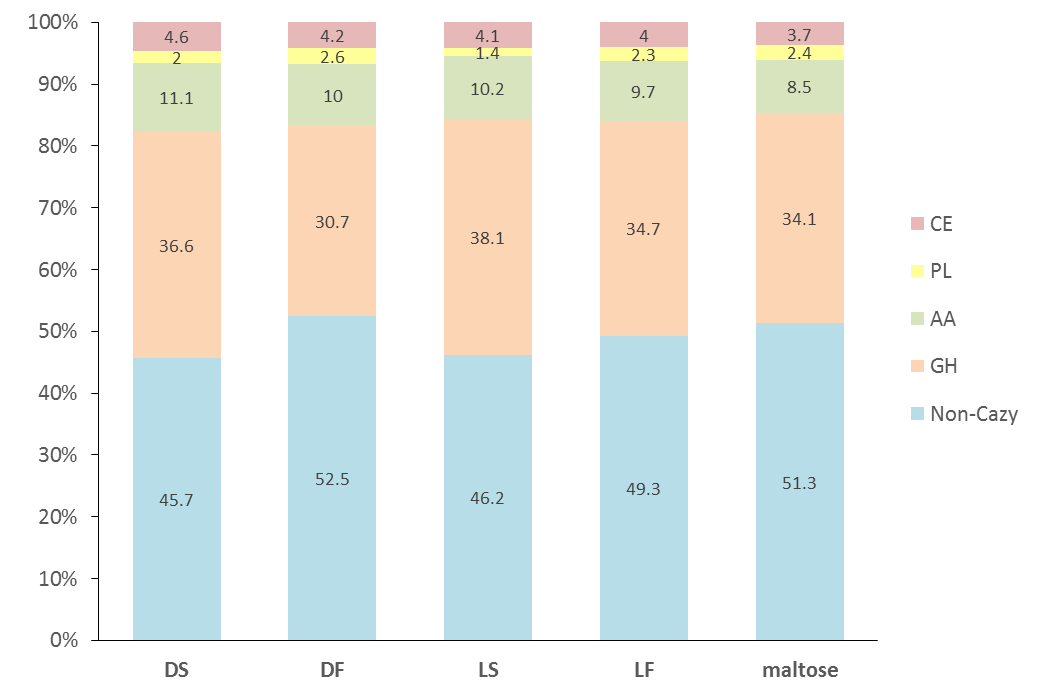
**

**Additional Figure S2** **Protein distribution in the secretomes of *P. cinnabarinus* grown on distilled straws, and maltose (reference).** GH: glycosyl hydrolases, AA: auxiliary activities, PL: polysaccharide lyases, CE: carbohydrate esterases. DS: lavandin stem straw; DF: lavandin flower straw; LS: lavender stem straw; LF: lavender flower straw

**Additional Table:**

**Additional Table S1** **Distribution of Non-CAZyme proteins identified in the secretomes of *P. cinnabarinus* grown on distilled straws**

|  | DS^a^ | DF^a^ | LS^a^ | LF^a^ | Reference (maltose) |
| --- | --- | --- | --- | --- | --- |
| Protein and amino acid metabolism enzymes | 38.0% | 36.1% | 41.7% | 25.9% | 37.5% |
| Lipid metabolism enzymes | 2.0% | 6.9% | 2.1% | 5.2% | 3.1% |
| Others | 18.0% | 15.3% | 20.8% | 22.4% | 10.9% |
| Unknown function proteins | 42.0% | 41.7% | 35.4% | 46.6% | 48.4% |

^a^ DS: lavandin stem straw; DF: lavandin flower straw; LS: lavender stem straw; LF: lavender flower straw
